# Two novel prognostic models for ovarian cancer respectively based on ferroptosis and necroptosis

**DOI:** 10.1186/s12885-021-09166-9

**Published:** 2022-01-17

**Authors:** Yang Li, Xiaojin Gong, Tongxiu Hu, Yurong Chen

**Affiliations:** 1 Department of Obstetrics and Gynecology, Tianjin Hospital, Tianjin, 300211 China; 2 Department of Oncology, Zhuji People’s Hospital of Zhejiang Province, Zhuji, 311800 Zhejiang China

**Keywords:** Ovarian cancer, Prognostic model, Ferroptosis, Necroptosis, Immune microenvironment

## Abstract

**Background:**

Platinum-resistant cases account for 25% of ovarian cancer patients. Our aim was to construct two novel prognostic models based on gene expression data respectively from ferroptosis and necroptosis, for predicting the prognosis of advanced ovarian cancer patients with platinum treatment.

**Methods:**

According to the different overall survivals, we screened differentially expressed genes (DEGs) from 85 ferroptosis-related and 159 necroptosis-related gene expression data in the GSE32062 cohort, to establish two ovarian cancer prognostic models based on calculating risk factors of DEGs, and log-rank test was used for statistical significance test of survival data. Subsequently, we validated the two models in the GSE26712 cohort and the GSE17260 cohort. In addition, we took gene enrichment and microenvironment analyses respectively using limma package and GSVA software to compare the differences between high- and low-risk ovarian cancer patients.

**Results:**

We constructed two ovarian cancer prognostic models: a ferroptosis-related model based on eight-gene expression signature and a necroptosis-related model based on ten-gene expression signature. The two models performed well in the GSE26712 cohort, but the performance of necroptosis-related model was not well in the GSE17260 cohort. Gene enrichment and microenvironment analyses indicated that the main differences between high- and low- risk ovarian cancer patients occurred in the immune-related indexes, including the specific immune cells abundance and overall immune indexes.

**Conclusion:**

In this study, ovarian cancer prognostic models based on ferroptosis and necroptosis have been preliminarily validated in predicting prognosis of advanced patients treated with platinum drugs. And the risk score calculated by these two models reflected immune microenvironment. Future work is needed to find out other gene signatures and clinical characteristics to affect the accuracy and applicability of the two ovarian cancer prognostic models.

**Supplementary Information:**

The online version contains supplementary material available at 10.1186/s12885-021-09166-9.

## Background

Ovarian cancer is a gynecological malignancy with the highest mortality, and ranks the fifth leading cause of cancer-related death in the USA [1]. In the USA, Ovarian cancer accounts for 2.38% of all female malignancies and 4.89% of all female cancer deaths, and the 5-year relative survival is only 48.6% [2]. The main reason for the high mortality rate from ovarian cancer is 75% of cases are already at advanced stage when diagnosed [3]. But early detection of ovarian cancer is difficult due to the insidious onset, including elvictor abdominal pain, early satiety, urinary frequency, constipation and abdominal distension [4]. Similar to other cancers, metastatic disease is the main cause to ovarian cancer related deaths [5]. In ovarian cancer, platinum–taxanes combination chemotherapy is a regular treatment after surgical cytoreduction [6]. However, most patients eventually relapse due to the strong drug resistance, especially for platinum drugs [7].

Drug resistance is a major problem in cancer treatment, leading to cell tolerance and failure in response to one or multiple agents. Since 25% of ovarian cancer patients are typically platinum resistant [8], it is necessary to predict platinum efficacy to each patient before chemotherapy. According to the previous studies, platinum resistance involved many biological processes in ovarian cancer, including altered drug metabolism, role of membrane transporters, dysregulation of cellular metabolism, cell death inhibition, DNA damage repair, long non-coding RNAs, epigenetics, oxidative stress [9]. Among them, cell death inhibition is the final reason to make the ovarian cancer cell resistant to platinum [10]. Therefore, identification of ovarian cancer related factors in cell death pathways is an effective way to predict the prognosis after platinum drug.

As the main cell death type, apoptosis has been well studied in ovarian cancer, but the major drug resistance is apoptosis resistance [11]. Ferroptosis and necroptosis are two newly discovered types of regulated necrosis, and a growing number of anti-cancer drugs have been reported to function as the activators of ferroptosis or necroptosis [12–14]. Ferroptosis is an iron-catalyzed form of regulated necrosis that functions through excessive peroxidation, recent studies have found that iron ptosis plays an important role in the occurrence and development of ovarian cancer [15]. Necroptosis is defined as a regulated necrosis type that requires the receptor interacting protein kinase 3 (RIPK3) and mixed lineage kinase like (MLKL), and is induced by death-related receptors, sensors and other mediators. It has been proved that both ferroptosis and necroptosis play important roles in cancer cells, especially in drug resistance [16, 17]. In ovarian cancer stem-like cells necroptosis was found driven by ALDH1A family selective inhibitors, which were broadly linked with resistance to chemotherapeutics such as paclitaxel and doxorubicin [18]. However, the relationship between ferroptosis- and necroptosis-related genes and prognosis of ovarian cancer patients is still vastly unknown, making it still a challenge for predicting prognosis of chemotherapy and developing novel therapies for ovarian cancer. Therefore, identifying cancer-related genes in ferroptosis and necroptosis is a promising way to predict prognosis of ovarian cancer patients using platinum drugs.

In this study, we established two prognostic models in ovarian cancer, involving ferroptosis and necroptosis respectively, based on the expression data from ovarian cancer patients with platinum drug in the GSE32062 cohort. Both two models were validated in the GSE26712 cohort, but the necroptosis-related model performed inappropriately in the GSE17260 cohort. Finally, we performed functional enrichment analysis and tumor microenvironment analysis to explore the molecular mechanisms that may influence the prognosis of ovarian cancer. Taken together, ferroptosis and necroptosis are two important pathways related to ovarian cancer prognosis, which can be used to build the prognostic prediction models under specific genetic backgrounds and platinum drug therapy.

## Materials and methods

### Data collection

#### The GSE32062 cohort, the GSE26712 cohort and the GSE17260 cohort

The ovarian cancer expression data and clinical data of GSE32062 cohort [19], GSE26712 cohort [20] and GSE17260 cohort [21] were downloaded from gene omnibus expression (GEO) database (https://www.ncbi.nlm.nih.gov/gds/). The expression data of GSE32062 cohort, GSE26712 cohort and GSE17260 cohort were produced from agilent whole human genome oligo microarray, affymetrix human genome U133A array, and Agilent-014850 whole human genome microarray 4x44K G4112F, respectively. In GSE32062 cohort, 10 cases were excluded due to the different platform, and the other 260 ovarian cancer patients with platinum drugs were included. In the GSE26712 cohort, all 185 late-stage (III/IV) ovarian cancer patients with platinum drugs were included as validation group 1. In GSE17260 cohort, 110 stage III/IV serious ovarian cancer patients who underwent platinum chemotherapy were included as validation group 2.

### Gene set

The ferroptosis-related gene set including 60 genes from references [22] and 41 genes from ferroptosis-related Kyoto Encyclopedia of Genes and Genomes (KEGG) pathway, among which 16 genes overlap and finally a total of 85 genes were included. All of 159 genes from necroptosis-related literatures and KEGG pathway were included as the necroptosis-related gene set in this study [23–25].

### Construction and validation prognostic models

Based on overall survival (OS), univariate and multivariate Cox regression analysis were used to screen prognosis-related genes respectively from ferroptosis-related gene set and necroptosis-related gene set. *P* value < 0.1 was the threshold for the entry from single factor into multivariate analysis, and stepwise regression was used for gene screening in multifactor analysis. The risk scores were calculated according to the normalized expression level of each screened gene and its corresponding regression coefficients. Patients were divided into high and low risk groups based on the median value of the risk score. Survival (version: 3.2–3) package and SurvMiner (version: 0.4.8) package were used for drawing survival curve, and log-rank test method was used for statistical significance test of survival data.

### Enrichment analysis

Differentially expressed genes (DEGs) were screened between high and low risk groups by limma package, and the screening threshold was |log_2_FC| > 2 and *P* value < 0.05. The screened DEGs were enriched in gene ontology (GO) and KEGG pathways by DAVID (https://david.ncifcrf.gov/).

### Immune microenvironment analysis

Relative abundance of each immune cell was calculated by GSVA (version: 1.32.0) package. Immune infiltration related indexes were calculated by ESTIMATE, including immune score, stromal score and ESTIMATE score. Correlation analysis between immune-related indexes and ferroptosis/necroptosis-related genes was conducted using psych (version: 2.0.8) package and drawn by corrplot (version: 0.84) package. Differential immune indexes between high and low risk groups were analyzed and drawn using stats (version: 3.6.2) package and ggpubr (version: 0.4.0) package.

### Statistical analysis

Student’s t-test was used to compare gene expression and immune related indexes between high and low risk groups. Log-rank test was used to compare OS between different groups by Kaplan-Meier analysis. Univariate and multivariate Cox regression analyses were applied using survival (version: 3.2–3) and glmnet (version: 4.0–2) packages in R, *P* < 0.1 was the threshold. All statistical analyses were conducted in R software. All *P* values were two-tailed, and less than 0.05 was considered statistically significant.

## Results

**Fig. 1:**
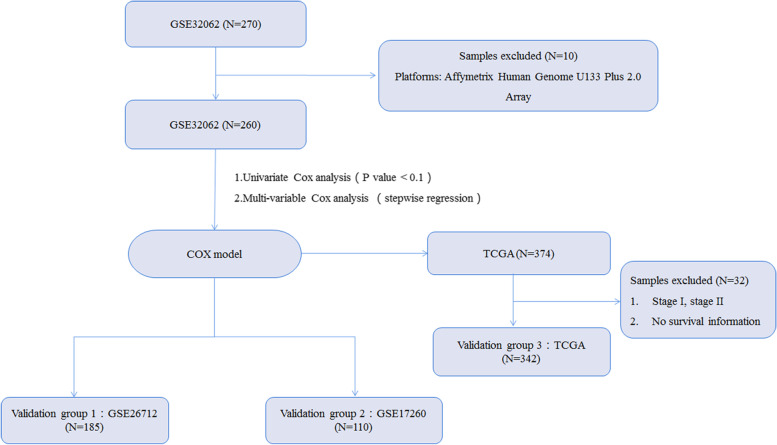
Flow chart of data collection

The work flow of this study is shown in Fig. 1. First, a total of 260 stage III/IV ovarian cancer patients with platinum treatment from GSE32062 were enrolled to construct a Cox model to predict the prognosis. Second, a total of 185 and 110 stage III/IV ovarian cancer patients with platinum treatment respectively from GSE26712 and GSE17260 were used as the validation group (validation group 1 and 2) to verify the models. The basic clinical characteristics of enrolled patients are listed in Table 1.

**Table 1:** Clinical characteristics of patients.

Cohort		GSE32062	GSE26712	GSE17260
Total number		260	185	110
Age (mean ± SD)		58.2 ± 10.8	62 ± 12	59 ± 12.6
Histology	Serous	260 (100%)	166 (89.73%)	110 (100%)
	Others	0	19 (10.27%)	0
Stage	Stage I	0	0	0
	Stage II	0	0	0
	Stage III	204 (78.46%)	144 (77.84%)	93 (84.55%)
	Stage IV	56 (21.54%)	41 (22.16%)	17 (15.45%)
Grade	Grade 1	0	0	26 (23.64%)
	Grade 2	131 (50.38%)	40 (21.62%)	41 (37.27%)
	Grade 3	129 (49.62%)	144 (77.84%)	43 (39.09%)
	Grade 4	0	1 (0.54%)	0
Treatment	Platinum	260 (100%)	185 (100%)	110 (100%)
	Taxane	260 (100%)	185 (100%)	110 (100%)
OS (median (interquartiles))		41.5 (1128) months	3.19 (0.06,13.65) years	30.5 (1,81) months

*OS* overall survival, *SD* standard deviation

### Identification of the prognostic ferroptosis-related and necroptosis-related genes in the GSE32062 cohort

**Fig. 2:**
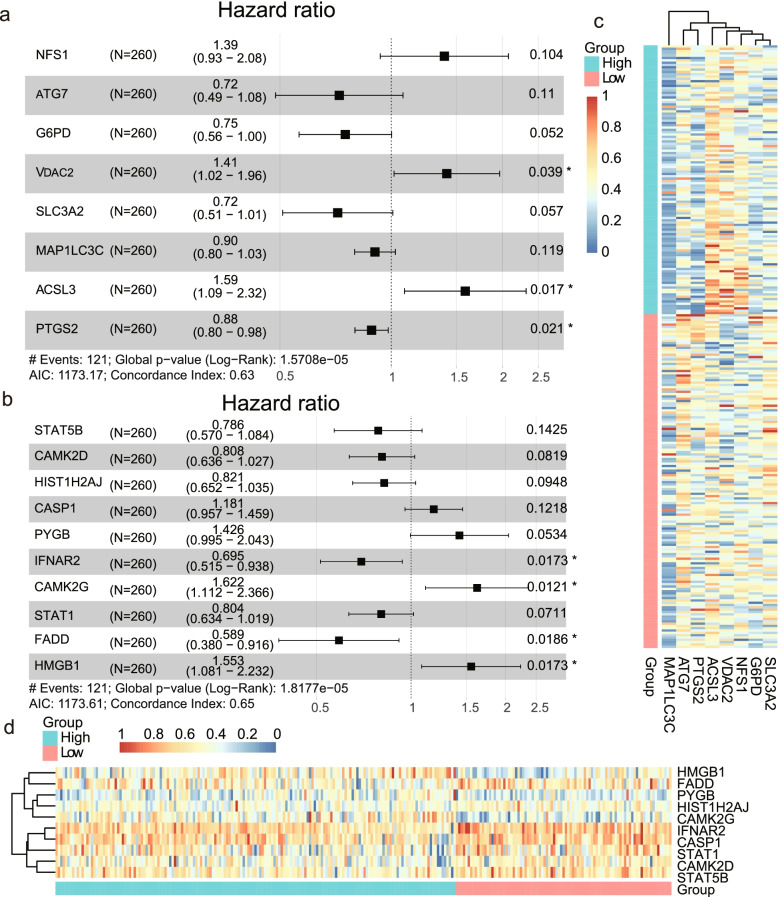
Prognostic models for ovarian cancer constructed in GSE32062. **a** Hazard ratio of genes included in the ferroptosis-related prognostic model. **b** Hazard ratio of genes included in the necroptosis-related prognostic model. **c** Heatmap of gene expression in the ferroptosis-related prognostic model. **d** Heatmap of gene expression in the necroptosis-related prognostic model

A total of 85 ferroptosis-related genes and a total of 159 necroptosis-related genes were used to analyze the relationship with prognosis of ovarian cancer patients in the GSE32062 cohort, respectively. Based on the expression profile, through the univariate and multivariate Cox regression analyses with OS, eight DEGs related to ferroptosis were finally identified closely related to OS, including *NFS1*, *ATG7*, *G6PD*, *VDAC2*, *SLC3A2*, *MAP1LC3C*, *ACSL3*, and *PTGS2* (Fig. 2a). Through the same method, we screened 10 necroptosis-related DEGs associated with OS of ovarian cancer patients, namely *STAT5B*, *CAMK2D*, *HIST1H2AJ*, *CASP1*, *PYGB*, *IFNAR2*, *CAMK2G*, *STAT1*, *FADD*, and *HMGB1* (Fig. 2b). The functions and ovarian cancer-related research of these genes are listed in Supplementary Table S1.

### Construction of four ferroptosis-related and necroptosis-related prognostic models in the GSE32062 cohort

Based on the identified DEGs, we constructed two prognostic models for ovarian cancer through Cox regression analyses. The risk score was calculated by the DEGs (n_(ferroptosis)_ = 8, n_(necroptosis)_ = 10), and the Yoden index was used to calculate the best cutoff value to divide the enrolled ovarian cancer patients into high and low risk groups (Fig. 2c, d). The characteristics of two models were described as follows.

#### Ferroptosis-related prognostic model

**Fig. 3:**
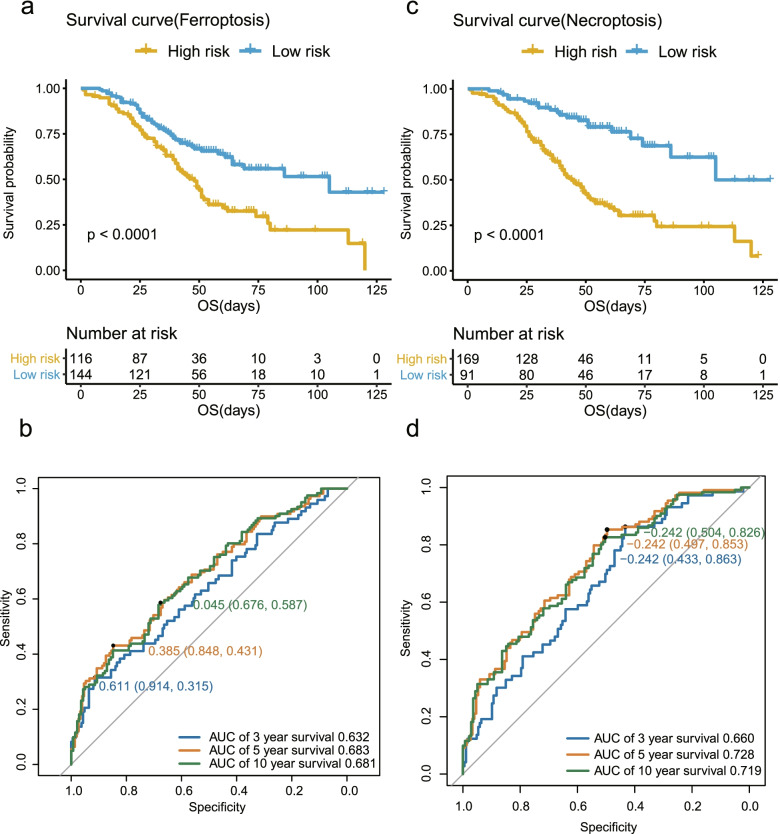
Prognostic analysis of the two models in the GSE32062 cohort. Survival curve (**a**) and ROC curves of 3, 5, 10 year survival (**b**) of high and low risk groups in the ferroptosis-related prognostic model in the GSE32062 cohort. Survival curve (**c**) and ROC curves of 3, 5, 10 year survival (**d**) of high and low risk groups in the necroptosis-related prognostic model in the GSE32062 cohort

We used eight screened DEGs to construct the ferroptosis-related prognostic model with OS. The OS of ovarian cancer patients prolonged with the high-expression of *ATG7*, *G6PD*, *SLC3A2*, *MAP1LC3C* and *PTGS2*, but shrank with the increased expression of *NFS1*, *VDAC2*, *ACSL3* (Fig. 2a). According to the best cutoff value of risk score, we divided 260 enrolled ovarian cancer patients into high-risk group (*n* = 116) and low-risk group (*n* = 144), shown in Fig. 2c. Survival analysis showed that the OS of high-risk group was significantly shorter than low-risk group (*P* < 0.0001; Fig. 3a). The risk score for OS was calculated by the predictive performance of the eight-gene receiver operating characteristic (ROC) curves. The area under the curve (AUC) reached 0.632 of 3-year survival, 0.683 of 5-year survival, and 0.681 of 10-year survival (Fig. 3b).

#### Necroptosis-related prognostic model

To build the necroptosis-related OS prognostic model, we used ten selected DEGs of *STAT5B*, *CAMK2D*, *HIST1H2AJ*, *CASP1*, *PYGB*, *IFNAR2*, *CAMK2G*, *STAT1*, *FADD* and *HMGB1*.The DEGs of *STAT5B*, *CAMK2D*, *HIST1H2AJ*, *IFNAR2*, *STAT1* and *FADD* were identified as six protective factors to this model, and *CASP1*, *PYGB*, *CAMK2G*, and *HMGB1* were identified as four risk factors (Fig. 2b). Survival analysis also showed a low-risk group was much longer than high-risk group in OS (*P* < 0.0001; Fig. 3c). The AUC of the ten-gene ROC curves reached 0.660 of 3-year survival, 0.728 of 5-year survival, and 0.719 of 10-year survival (Fig. 3d).

### Validation of the prognostic models in the GSE26712 cohort

**Fig. 4:**
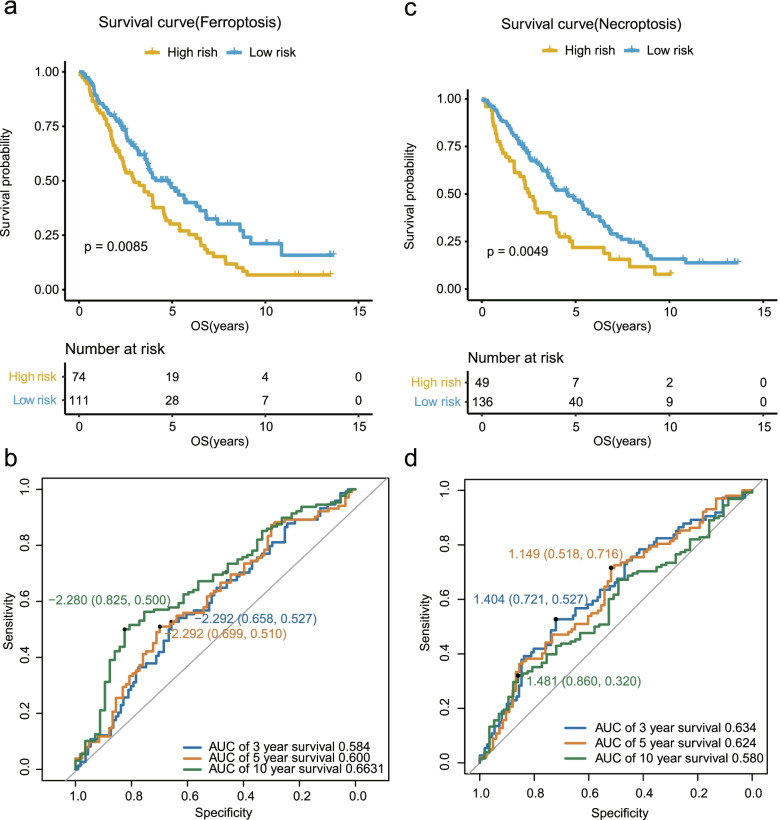
Performance of the two models in the GSE26712 cohort. Survival curve (**a**) and ROC curves (**b**) of high and low risk groups in the ferroptosis-related prognostic model in the GSE26712 cohort. Survival curve (**c**) and ROC curves (**d**) of high and low risk groups in the necroptosis-related prognostic model in the GSE26712 cohort

Here, we used GSE26712 cohort to validate the ferroptosis-related and necroptosis-related prognostic models with OS. The AUC of ferroptosis-related ROC was 0.584 of 3-year survival, 0.600 of 5-year survival, 0.663 of 10-year survival (Fig. 4b). And the AUC of necroptosis-related ROC was 0.634 of 3-year survival, 0.624 of 5-year survival, 0.580 of 10-year survival (Fig. 4d).

Following the same formula of risk score from GSE32062 cohort, we calculated risk score of each patient enrolled from the GSE26712 cohort. According to the risk scores, the GSE26712 cohort were divided into high-risk group and low-risk group. The OS of high-risk group was significantly shorter than low-risk group in ferroptosis-related prognostic model (*P* = 0.0085; Fig. 4a), and the similar result was also shown in necroptosis-related prognostic model (*P* = 0.0049; Fig. 4c). Therefore, the ferroptosis- and the necroptosis-related prognostic models with OS for ovarian cancer also worked well in the GSE26712 cohort.

### Validation of the prognostic models in the GSE17260 cohort

**Fig. 5:**
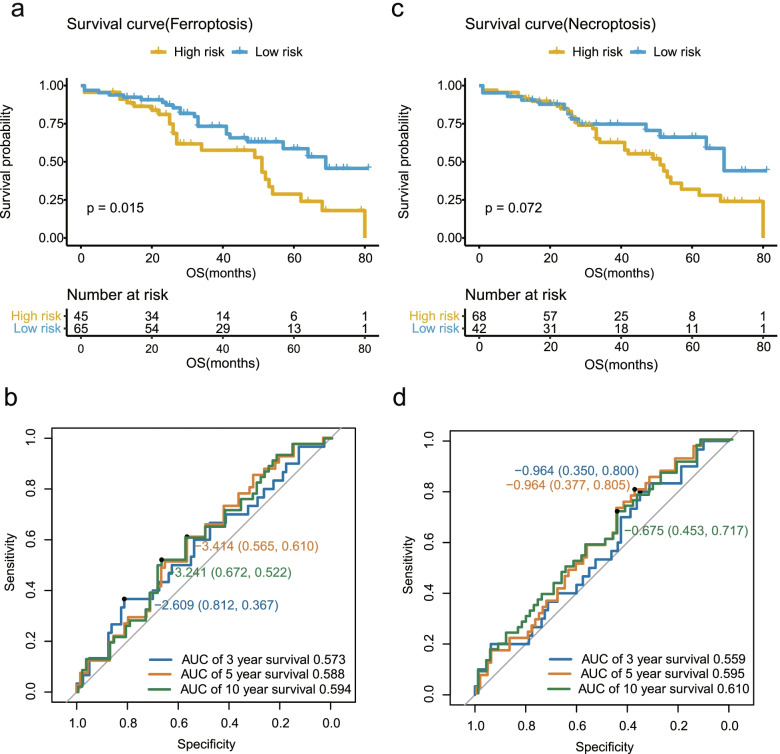
Performance of the two models in the GSE17260 cohort. Survival curve (**a**) and ROC curves (**b**) of high and low risk groups in the ferroptosis-related prognostic model in the GSE17260 cohort. Survival curve (**c**) and ROC curves (**d**) of high and low risk groups in the necroptosis-related prognostic model in the GSE17260 cohort

To ensure the robustness of the two models in our study, a total of 110 ovarian cancer patients from the GSE17260 cohort were enrolled for further validation. The patients were clustered into high-risk group and low-risk group following the same way used in the GSE32062 cohort. The AUC of 3-, 5- and 10-year survival were 0.573, 0.588 and 0.594 in ferroptosis-related ROC (Fig. 5b), and 0.559, 0.595, 0.610 respectively in necroptosis-related ROC (Fig. 5d). Meanwhile, the survival curve showed significant differences between high and low risk groups in ferroptosis-related model (*P* = 0.015; Fig. 5a), but no statistical significance in the necroptosis-related model (*P* = 0.072; Fig. 5c). The results revealed that the ferroptosis-related prognostic model with OS for ovarian cancer worked well in the GSE17260 cohort, but the necroptosis-related model should be further optimized in the future.

### Functional enrichment analysis in the GSE32062 cohort

**Fig. 6:**
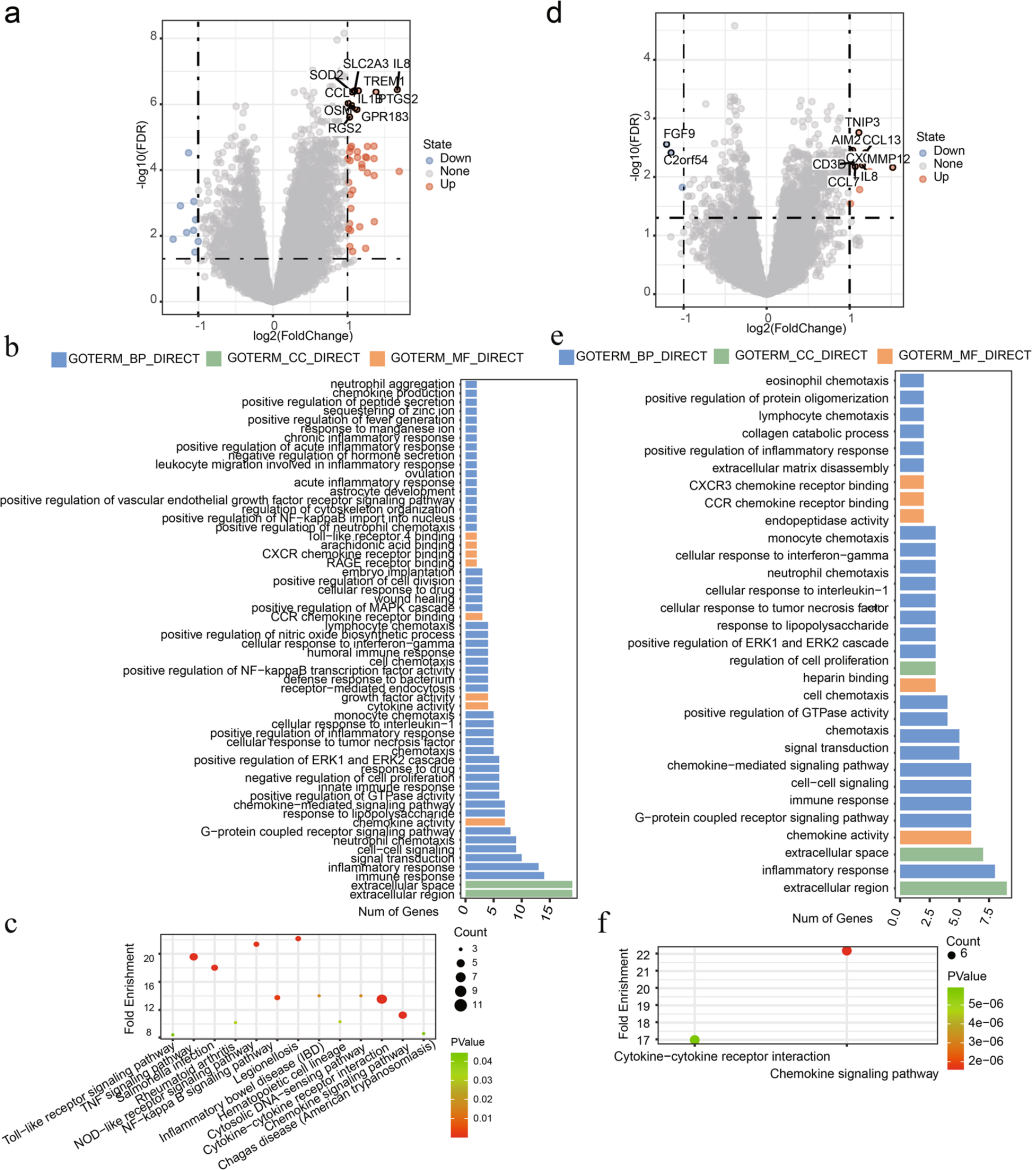
Functional analysis in the GSE32062 cohort. In the ferroptosis-related prognostic model: **a**: DEGs between high- and low-risk groups. **b**: GO analysis. **c**: KEGG analysis. In the necroptosis-related prognostic model: **d**: DEGs between high- and low-risk groups. **e**: GO analysis. **f**: KEGG analysis

To explore the biological functions and regulatory pathways related to the prognostic models, we selected the DEGs between the high-risk group and low-risk group to conduct GO and KEGG analyses. Interestingly, many immune related functions and pathways were enriched in both ferroptosis-related and necroptosis-related risk models (Fig. 6a, d).

In the ferroptosis-related prognostic model, 10 immune-related GO terms were significantly enriched in (*P* < 0.05), including five biological processes (BPs) of immune response, humoral immune response, innate immune response, chemokine-mediated signaling pathway, positive regulation of NF-κB transcription factor activity; and five immune-related molecular functions (MFs) of chemokine activity, cytokine activity, Toll-like receptor 4 binding, CCR chemokine receptor binding and CXCR chemokine receptor binding (Fig. 6b). Besides, six immune-related KEGG pathways were also enriched in the ferroptosis-related prognostic model, including cytokine-cytokine receptor interaction, chemokine signaling pathway, TNF signaling pathway, NF-kappa B signaling pathway, NOD-like receptor signaling pathway and Toll-like receptor signaling pathway (Fig. 6c).

In the necroptosis-related prognostic model, two immune-related BPs were enriched, including chemokine-mediated signaling pathway and immune response; three immune-related MFs were enriched, including chemokine activity, CCR chemokine receptor binding and CXCR3 chemokine receptor binding; two immune-related KEGG pathways were enriched, including chemokine signaling pathway and cytokine-cytokine receptor interaction (Fig. 6e, f).

### Microenvironment analysis in the GSE32062 cohort

According to the functional enrichment analysis, immune-related factors were identified as key elements to ovarian cancer patients in the GSE32062 cohort. To comprehensively analyze the importance of the immune-related factors in the two above prognostic models, 45 immune-related indexes calculated from the gene expression data were included.

**Fig. 7:**
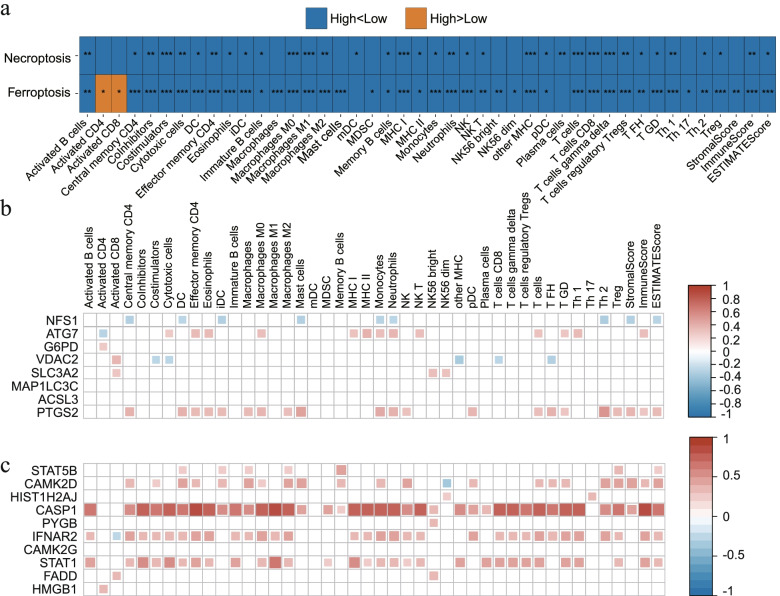
Microenvironment analysis in the GSE32062 cohort. **a**: Comparison between the high- and low-risk groups in the immune-related indexes of the two models. Blue: the value of the high-risk group is higher than the low-risk group; Orange: the value of the high-risk group is lower than the low-risk group. **b**: Correlation analysis between gene expression and immune-related indexes in the ferroptosis-related prognostic model. **c**: Correlation analysis between gene expression and immune-related indexes in the necroptosis-related prognostic model. *: *P* < 0.05; **: *P* < 0.01; ***: *P* < 0.001

In the ferroptosis-related prognostic model, 43 of the 45 immune-related factors had significant relationship with risk score, and plasma cell and mDC were the two exceptions. As expected, almost all the immune-related factors had negative relationships with risk scores, except activated CD4 and activated CD8 (Fig. 7a). For the 10 DEGs enrolled in the ferroptosis-related prognostic model, we comprehensively analyzed the correlation between each DEG expression and immune-related factors. *NFS1*, *ATG7*, *VDAC2* and *PTGS2* were four genes associated with more than five factors. *NFS1* and *VDAC2* were two DEGs that were negatively associated with immune-related factors, involving 9 and 5 factors, respectively. *ATG7* and *PTGS2* were positively associated with 13 and 21 immune-related factors, respectively (Fig. 7b).

In the necroptosis-related prognostic model, 35 of 45 immune-related factors had significant relationship with risk score, and all of them showed positive correlation (*P* < 0.05; Fig. 7a). For the 12 DEGs enrolled in this model, except CAMK2D-NK56 dim and IFMAR2-activated CD8, all the significant relationships between each gene and factors were positive. For the correlation analysis of each DEG with immune-related factors, five DEGs were associated with more than 30 immune-related factors, including *CASP1*, *IFNAR2*, *STAT1*, *CYLD* and *STAT2*. Among them, *CASP1* was a DEG most associated with immune-related factors, involving 40 factors. On the contrary, five DEGs were associated with less than 2 immune-related factors, including *HIST1H2AJ*, *PYGB*, *CAMK2G*, *FADD* and *HMGB1*. *CAMK2G* was a DEG associated with none of the 45 immune-related factors (Fig. 7c).

## Discussion

In this study, we constructed two ovarian cancer prognostic models based on the GSE32062 cohort, related to ferroptosis and necroptosis, respectively. The ferroptosis-related prognostic model performed well in both of the two validation groups, but the necroptosis-related model was validated in only one cohort (Figs. 4, 5). Both functional analysis and microenvironment analysis showed that immune-related factors had the close relationship with the risk score in the two prognostic models.

Although the current standard treatment is primary surgery followed by platinum-based chemotherapy, there is still a significant proportion of patients with platinum-resistant ovarian cancer [26]. Recent studies reported that ferroptosis and necroptosis are two important pathways to affect the efficacy of platinum drugs [12–14], which may further affect the prognosis of ovarian cancer patients treated with platinum drugs. In this study, we established two ovarian cancer prognostic models based on gene expression data respectively of ferroptosis and necroptosis pathways, and these two prognostic models performed well in both the modeling cohort of GSE32062 and the validation cohort of GSE26712 (Figs. 3, 4), and the ferroptosis-related model performed well in the GSE17260 cohort (Fig. 5a-b).

Besides, although the necroptosis-related prognostic model cannot be applied in the GSE17260 cohort, the survival probability after 36 months were obviously different between high and low risk groups (Fig. 5c). These results suggested that some clinical indicators, as well as expression levels of genes apart from the gene set used in this study, need to be taken into consideration. Studies have found that some clinical characteristics, such as age, performance status, FIGO stage, residual disease, histology, and BRCA/HRD, are the potential prognostic factors of ovarian cancer [27–29]. The model constructed by neglecting these factors may not be suitable for some patients. However, due to the use of common data from GEO database, these cases lack many clinical indicators and cannot be used for multivariate analysis, which is also a shortcoming of this study. The models should be further optimized based on prospective clinical study to improve their clinical usefulness.

As immune infiltrate in the microenvironment plays a key role in ovarian cancer development [30], combined with the enrichment results of many immune-related genes in the current study, we conducted a comprehensive correlation analysis to find out which immune cells were involved in the two prognostic models. The risk scores of the two ovarian cancer prognostic models had the negative correlation with ESTIMATE score, immune score and stromal score, as well as with the relative abundance of most immune cells (Fig. 7a). Among them, activated CD4 and activated CD8 were only two types of immune cells that could promote the ovarian cancer progress in the ferroptosis-related pathway, and similar results were also reported in the previous studies [31, 32]. However, in the necroptosis-based prognostic model, all involved immune-related indexes in this study showed a negative relationship with risk scores. These results indicated that the abundance of activated CD4 and activated CD8 could be used as a key biomarker to distinguish whether ferroptosis or necroptosis plays a dominant role in an ovarian cancer patient.

Although we display a series of significant results, there are still some limitations in this study. First, the included genes of ferroptosis and necroptosis in this study are mainly based on previous studies, so that some unreported related genes may be ignored and excluded. This problem would reduce the accuracy and applicability of the prognostic models. Second, the lack of clinical information to build the prognostic models may miss by key prognostic factors. Third, the two prognostic models constructed for ovarian cancer patients in this study need to verification in the clinical practice. According to the above, future work will focus on the two points: (1) Screening novel genes related to ferroptosis and necroptosis in more cohorts; (2) Collecting adequate ovarian cancer cases and clinical information to validate and optimize the two prognostic models, and compare them with the clinical gold standard, so as to make sure that the prognosis models are useful for clinical practice.

## Conclusions

In conclusion, based on ferroptosis and necroptosis, our study constructed two ovarian cancer prognostic models for predicting the prognosis of advanced ovarian cancer patients treated with platinum drugs. The two models proved closely related to tumor immunity. Further studies are needed to optimize the two models by enrolling in other related genes and clinical information.

## Supplementary Information

**Additional file 1:** **Supplementary Table S1.** Main functions and researches of genes identified in the prognosis signatures^#^.

## Data Availability

The datasets generated and/or analyzed during the current study are available in the GEO repository. (https://www.ncbi.nlm.nih.gov/gds/).

## Abbreviations

RIPK3: Receptor interacting protein kinase 3
MLKL: Mixed lineage kinase like
KEGG: Kyoto Encyclopedia of Genes and Genomes
OS: Overall survival
DEGs: Differentially expressed genes
GO: Gene ontology
ROC: Receiver operating characteristic
AUC: Area under the curve
BPs: Biological processes
MFs: Molecular functions
