# Supplementary material for: Two novel prognostic models for ovarian cancer respectively based on ferroptosis and necroptosis
Source: BMC Cancer. 2022 Jan 17;22:74. doi: 10.1186/s12885-021-09166-9 (PMC8764839; doi:10.1186/s12885-021-09166-9)
Supplement: Supplementary file 1 — Additional file 1: Supplementary Table S1. Main functions and researches of genes identified in the prognosis signatures#. [file 12885_2021_9166_MOESM1_ESM.docx]

**Supplementary Table S1** Main functions and researches of genes identified in the prognosis signatures^#^

| **Gene** | **Protein** | **Pathways** | **Function** | **Study in OC** |
| --- | --- | --- | --- | --- |
| **Ferroptosis-related prognostic model** | | | | |
| *ATG7* | Encode an E1-like activating enzyme | Class I MHC mediated antigen processing and presentation  Innate Immune System  Macroautophagy  HIV Life Cycle  Oncogenic MAPK signaling  Autophagy pathway  Ferroptosis  Senescence and Autophagy in Cancer | Cytoplasm to vacuole transport; autophagic death induced by caspase-8 inhibition; essential for autophagy and cytoplasmic to vacuole transport, and modulate p53-dependent cell cycle pathways during prolonged metabolic stress. | Hu et al. ^[1]^  Chen et al. ^[2]^  Tang et al. ^[3]^ |
| *G6PD* | Encode glucose-6-phosphate dehydrogenase | Pentose phosphate pathway  Metabolism  Gene Expression  Central carbon metabolism in cancer  Regulation of TP53 Activity  NRF2 pathway | Catalyzes the first and rate-limiting step of the oxidative branch within the pentose phosphate pathway/shunt. | Feng et al. ^[4]^ |
| *SLC3A2* | Encode a cell surface, transmembrane protein | Transport of glucose and other sugars, bile salts and organic acids, metal ions and amine compounds  Histidine, lysine, phenylalanine, tyrosine, proline and tryptophan catabolism  Glucose / Energy Metabolism  Ferroptosis  mTOR signaling pathway (KEGG)  Viral mRNA Translation | Component of several heterodimeric amino acid transporter complexes. | Cui et al. ^[5]^  Huang et al. ^[6]^ |
| *MAP1LC3C* | Encode autophagy related protein | Ferroptosis  Cellular Senescence (REACTOME)  Macroautophagy  Senescence and Autophagy in Cancer | Ubiquitin-like modifier that plays a crucial role in antibacterial autophagy (xenophagy) through the selective binding of CALCOCO2. May also play a role in aggrephagy, the macroautophagic degradation of ubiquitinated and aggregated proteins. | —— |
| *PTGS2* | Encodes prostaglandin-endoperoxide synthase | Angiogenesis (CST)  NF-kappa B signaling  PI3K-Akt signaling pathway  IL-17 Family Signaling Pathways  MicroRNAs in cancer  Pathways in cancer  Toll-Like receptor Signaling Pathways | Key enzyme in prostaglandin biosynthesis, and acts both as a dioxygenase and as a peroxidase. Also responsible for the prostanoid biosynthesis involved in inflammation and mitogenesis. | Zhang et al. ^[7]^  Ye et al. ^[8]^  Barnard et al. ^[9]^  Shao et al. ^[10]^ |
| *NFS1* | Encode proteins belonging to the class-V family of pyridoxal phosphate-dependent aminotransferases | Metabolism of water-soluble vitamins and cofactors  Metabolism  Biosynthesis of cofactors  Sulfur relay system  Thiamine metabolism  Mitochondrial iron-sulfur cluster biogenesis | Catalyzes the removal of elemental sulfur from cysteine to produce alanine. Supplies the inorganic sulfur for iron-sulfur (Fe-S) clusters. May be involved in the biosynthesis of molybdenum cofactor. | —— |
| *VDAC2* | Encode a member of the voltage-dependent anion channel pore-forming family of proteins | Apoptotic Pathways in Synovial Fibroblasts  Cellular senescence (KEGG)  Ferroptosis  Necroptosis  Apoptosis and survival_Regulation of Apoptosis by Mitochondrial Proteins | The main pathway for metabolite diffusion across the mitochondrial outer membrane. Binding of ceramide promotes the mitochondrial outer membrane permeabilization (MOMP) apoptotic pathway. | Li et al. ^[11]^ |
| *ACSL3* | Encode an isozyme of the long-chain fatty-acid-coenzyme A ligase family | Fatty Acid Biosynthesis  PPAR signaling pathway  Peroxisome  Ferroptosis  Fatty acid biosynthesis (KEGG)  Mitochondrial LC-Fatty Acid Beta-Oxidation | Play a key role in lipid biosynthesis and fatty acid degradation. Has mainly an anabolic role in energy metabolism. | Yang et al. ^[12]^  Chen et al. ^[13]^ |
| **Necroptosis-related prognostic model** | | | | |
| *STAT5B* | Encode a member of the STAT family of transcription factors | Common Cytokine Receptor Gamma-Chain Family Signaling Pathways  Th17 cell differentiation  FGFR1 mutant receptor activation  Pathways in cancer | It is involved in diverse biological processes, such as TCR signaling, apoptosis, adult mammary gland development, and sexual dimorphism of liver gene expression. | Li et al. ^[14]^ |
| *CAMK2D* | Encode a member of serine/threonine protein kinase family and to the Ca(2+)/calmodulin-dependent protein kinase subfamily | RET signaling  ErbB signaling pathway  Activation of cAMP-Dependent PKA  Cytokine Signaling in Immune system  Calcium signaling pathway  cAMP signaling pathway  Necroptosis  Oocyte meiosis  Pathways in cancer  Proteoglycans in cancer  Wnt signaling pathway  MAPK Signaling: Oxidative Stress Pathway  ERK Signaling | Involved in the regulation of Ca(2+) homeostatis and excitation-contraction coupling (ECC) in heart by targeting ion channels, transporters and accessory proteins involved in Ca(2+) influx into the myocyte, Ca(2+) release from the sarcoplasmic reticulum (SR), SR Ca(2+) uptake and Na(+) and K(+) channel transport. Targets also transcription factors and signaling molecules to regulate heart function. | Xu et al. ^[15]^  Permuth-Wey et al. ^[16]^ |
| *HIST1H2AJ* | Encode a replication-independent histone that is a variant H2A histone | Activated PKN1 stimulates transcription of AR (androgen receptor) regulated genes KLK2 and KLK3  Cellular Senescence (REACTOME)  Cell Cycle, Mitotic  DNA Damage/Telomere Stress Induced Senescence  Necroptosis | Basic nuclear proteins that are responsible for the nucleosome structure of the chromosomal fiber in eukaryotes | —— |
| *IFNAR2* | Encode a type I membrane protein | Toll-like Receptor Signaling Pathway  Immune response IFN alpha/beta signaling pathway  PI3K-Akt signaling pathway  JAK-STAT signaling pathway  Viral mRNA Translation  PEDF Induced Signaling  Necroptosis  Cytokine Signaling in Immune system | Associates with IFNAR1 to form the type I interferon receptor. Receptor for interferons alpha and beta. Involved in IFN-mediated STAT1, STAT2 and STAT3 activation. Isoform 1 and isoform 2 are directly involved in signal transduction. | Takemoto et al. ^[17]^ |
| *STAT1* | Encode a member of the STAT protein family | Immune response IL-23 signaling pathway  Prolactin Signaling Pathway  JAK-STAT signaling pathway  FGFR1 mutant receptor activation  Necroptosis  Endometrial cancer  Pathways in cancer  Colorectal Cancer Metastasis  EGF/EGFR Signaling Pathway  Integrated Cancer Pathway | Mediate the expression of a variety of genes, play an important role in immune responses to viral, fungal and mycobacterial pathogens. Signal transducer and transcription activator that mediates cellular responses to interferons (IFNs), cytokine KITLG/SCF and other cytokines and other growth factors. | Liu et al. ^[18]^  Josahkian et al. ^[19]^  Zhang et al. ^[20]^  Wang et al. ^[21]^  Tian et al. ^[22]^  Li et al. ^[23]^  Au et al. ^[24]^ |
| *FADD* | Encode an adaptor molecule | TNFR1 Pathway  Apoptosis Modulation and Signaling  TNF signaling  Apoptosis and survival Caspase cascade  Necroptosis  Apoptosis and Autophagy  Pathways in cancer  Platinum drug resistance  ERK Signaling  Integrated Breast Cancer Pathway | Interacts with various cell surface receptors and mediates cell apoptotic signals. Recruits caspase-8 or caspase-10 to the activated Fas (CD95) or TNFR-1 receptors. Can be recruited by TNFRSF6/Fas-receptor, tumor necrosis factor receptor, TNFRSF25, and TNFSF10/ TRAIL-receptor, thus participates in the death signaling initiated by these receptors. | —— |
| *CASP1* | Encode a protein of a member of the cysteine-aspartic acid protease (caspase) family | TNFR1 Pathway  TP53 Regulates Transcription of Cell Death Genes  Gene Expression  Apoptosis Modulation and Signaling  VEGF Signaling  Apoptosis and survival Caspase cascade  MAPK signaling pathway | Involve in a variety of inflammatory processes by proteolytically cleaving other proteins into active mature peptides. Plays a key role in cell immunity as an inflammatory response initiator. Induce cell apoptosis and may function in various developmental stages | Feng et al. ^[25]^  Tremblay et al. ^[26]^ |
| *PYGB* | Encode a glycogen phosphorylase found predominantly in the brain | Activation of cAMP-Dependent PKA  Metabolism  Insulin resistance  Necroptosis  Glucagon signaling pathway | Catalyzes the rate-determining step in glycogen degradation. Regulates glycogen mobilization. | Zhou et al. ^[27]^  Zhang et al. ^[28]^ |
| *CAMK2G* | The product of this gene is one of the four subunits of an enzyme which belongs to the serine/threonine protein kinase family and the Ca(2+)/calmodulin-dependent protein kinase subfamily | RET signaling  Development Angiotensin activation of ERK  Transmission across Chemical Synapses  ErbB signaling pathway  ICos-ICosL Pathway in T-Helper Cell  Calcium signaling pathway  Oocyte meiosis  Proteoglycans in cancer | Calcium/calmodulin-dependent protein kinase that functions autonomously after Ca(2+)/calmodulin-binding and autophosphorylation, and is involved in sarcoplasmic reticulum Ca(2+) transport in skeletal muscle and may function in dendritic spine and synapse formation and neuronal plasticity. | —— |
| *HMGB1* | Encode a protein that belongs to the High Mobility Group-box superfamily | Necroptosis  Apoptotic cleavage of cellular proteins  Retinoblastoma (RB) in Cancer  Cytosolic sensors of pathogen-associated DNA  Innate Immune System  Autophagy – animal  Activated TLR4 signalling  Activation of DNA fragmentation factor  Chromatin Regulation / Acetylation  Telomere C-strand (Lagging Strand) Synthesis  Platinum Pathway, Pharmacokinetics /Pharmacodynamics  Autophagy Pathway  DNA Damage Response (only ATM dependent)  Advanced glycosylation endproduct receptor signaling | Replication, transcription, chromatin remodeling, V(D)J recombination, DNA repair and genome stability. In the cytoplasm functions as sensor and/or chaperone for immunogenic nucleic acids implicating the activation of TLR9-mediated immune responses, and mediates autophagy. Involved in oxidative stress-mediated autophagy. Involved in induction of immunological tolerance by apoptotic cells; its pro-inflammatory activities when released by apoptotic cells are neutralized by reactive oxygen species (ROS)-dependent oxidation specifically on Cys-106 | Wang et al. ^[29]^  Cámara-Quílez et al. ^[30, 31]^  Jiang et al. ^[32]^  Li et al. ^[33]^  Jiang et al ^[34]^.  Shu et al. ^[35]^  Waki et al. ^[36]^  Machado et al. ^[37]^  Ju et al. ^[38]^  Paek et al. ^[39]^  Zhou et al. ^[40]^  Wang et al. ^[41]^  Zhang et al. ^[42]^  Li et al. ^[43]^ |

^#^ The pathways and functions of the genes were obtained from GeneCards database (<http://www.genecards.org/>). OC, ovarian cancer
